# Zero-static power radio-frequency switches based on MoS_2_ atomristors

**DOI:** 10.1038/s41467-018-04934-x

**Published:** 2018-06-28

**Authors:** Myungsoo Kim, Ruijing Ge, Xiaohan Wu, Xing Lan, Jesse Tice, Jack C. Lee, Deji Akinwande

**Affiliations:** 1 Microelectronics Research Center, The University of Texas at Austin, Austin, TX 78758 USA; 2 NG Next, Northrop Grumman Corporation, One Space Park, Redondo Beach, Los Angeles, CA 90278 USA

## Abstract

Recently, non-volatile resistance switching or memristor (equivalently, atomristor in atomic layers) effect was discovered in transitional metal dichalcogenides (TMD) vertical devices. Owing to the monolayer-thin transport and high crystalline quality, ON-state resistances below 10 Ω are achievable, making MoS_2_ atomristors suitable as energy-efficient radio-frequency (RF) switches. MoS_2_ RF switches afford zero-hold voltage, hence, zero-static power dissipation, overcoming the limitation of transistor and mechanical switches. Furthermore, MoS_2_ switches are fully electronic and can be integrated on arbitrary substrates unlike phase-change RF switches. High-frequency results reveal that a key figure of merit, the cutoff frequency (*f*_c_), is about 10 THz for sub-μm^2^ switches with favorable scaling that can afford *f*_c_ above 100 THz for nanoscale devices, exceeding the performance of contemporary switches that suffer from an area-invariant scaling. These results indicate a new electronic application of TMDs as non-volatile switches for communication platforms, including mobile systems, low-power internet-of-things, and THz beam steering.

## Abstract (web summary)

The wide application of wireless communications in various technologies calls for the development of robust yet compact radio-frequency switches. Here, Kim et al. utilize MoS_2_ based non-volatile memristors to switch up to THz frequencies in sub µm^2^ areas, whilst the switches consume zero-static energy.

## Introduction

Worldwide advancement in wireless communication and connectivity systems for reconfigurable radios, mobile devices, internet-of-things (IoT), and phased array networks has resulted in an ever-increasing demand for radio-frequency (RF) switches, which can route signals from one band to another^1–10^. Conventional switches are realized with solid state diode or transistor devices^1–3^, which are volatile and as a consequence dissipate both dynamic and static energy. The former is due to a switching event and the latter a consequence of the required DC bias or hold voltage. To reduce leakage current, switches based on micro-electro-mechanical systems (MEMS) have been investigated^4–6^. However, MEMS devices require rather large switching voltages (~10–100 V) and are difficult to integrate onto arbitrary platforms due to complex fabrication and packaging.

For the purpose of improving energy efficiency, non-volatile switches are attractive because they require no hold voltage for operation and, as a benefit, consume zero-static power. Toward this end, non-volatile memory devices such as resistive random-access memory (RRAM) and phase-change memory (PCM) have been recently considered for RF switch applications. These memory devices afford resistance modulation between a high-resistance state (HRS, *R*_OFF_) and a low-resistance state (LRS, *R*_ON_) and subsequently retain the current state without power consumption^11,12^. RRAM devices are typically realized with amorphous transitional metal oxides that have LRS values >1 kΩ, making them unsuitable for RF switching due to system requirements for *R*_ON_ to be much <50 Ω in order to avoid excessive insertion losses. Moreover, a large forming voltage is typically required to initiate the RRAM device^11^. On the other hand, RF switches based on PCM have shown promising results with low *R*_ON_, high endurance, and decent *f*_c_ (cutoff frequency) figure of merit (FOM)^7–9^. However, they have fundamental limitations, including (1) the need for an integrated heater, (2) relatively slow switching times due to heat transport, and (3) unfavorable area dependency that compromises scaling to higher frequencies.

**Fig. 1:**
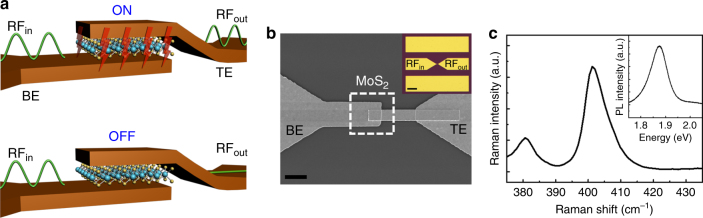
Device schematics and images with material characterization. **a** Simplified illustration of the signal transmission and device structure of the RF switches based on monolayer MoS_2_. **b** Zoomed-in plan view SEM image of a MoS_2_ RF switch with lateral area of 1 × 1 μm^2^. Scale bar, 2 μm. The dashed box in **b** marks the area covered with MoS_2_. The inset is a top-view optical image of a fabricated MoS_2_ RF switch with Au electrodes. Scale bar, 50 μm. **c** Raman and photoluminescence (inset) spectra of CVD-grown monolayer MoS_2_

Here we present forming-free MoS_2_ non-volatile memory^13^ as RF switches that overcome the aforementioned limitations of RRAM and PCM devices. LRS down to about 4 Ω due to thin transport layer enables low insertion loss for signal transmission (~0.3 dB) while maintaining sufficiently low capacitance (~20 fF/μm^2^) to achieve isolation at GHz and THz frequencies. Moreover, MoS_2_ RF switches are straightforward to realize with no need for a heater (Fig. 1), are programmable with a voltage around 1 V, afford fast switching times similar to ion transport in RRAM, and enjoy favorable scaling with area (*A*) for higher frequency capability. With regards to the latter, RF circuits for wideband or high frequency operation require an RF switch with low ON-state resistance and low OFF-state capacitance (*C*_OFF_) to obtain a high cutoff frequency FOM, *f*_c_ = 1/2*πR*_ON_*C*_OFF_. Notably, the novel combination of one-dimensional area invariant *R*_ON_ and two-dimensional (2D) area-dependent *C*_OFF_ yields an *f*_c_ that can be scaled to higher frequencies by simply reducing device area, a defining advantage over PCM switches^9,14^ where capacitance is proportional to width but *R*_ON_, inversely dependent, hence, forcing a compromise between frequency scalability and insertion loss. Experimentally, an area-normalized FOM of ~1 THz-μm^2^ has been realized with promising prospects for *f*_c_ >100 THz for device dimensions <0.01 μm^2^.

## Results

### Material characterization and device structure

Figure 1 shows the metal–insulator–metal (MIM) device structure and optical characterization of a monolayer MoS_2_ RF switch fabricated on a 100-μm-thick Corning Willow glass using electron beam lithography (EBL) and e-beam metal evaporation. The switch stack consists of 70 and 60 nm gold (Au) electrodes that serve as the top and bottom electrodes, respectively, both with 2 nm chromium (Cr) adhesion layers connecting the electrodes to the MoS_2_ sheet. All metal layers are formed by lift-off after patterning and the overlap between the electrodes defines the switch area. MoS_2_ atomic sheets are synthesized by chemical vapor deposition (CVD)^15,16^ and integrated onto the prepatterned bottom electrode using dry or wet transfer methods^17^. Figure 1a depicts the device structure allowing (ON) or rejecting (OFF) the transmission of RF signal depending on the switch direct current (DC) status. For RF measurements, ground–signal–ground (GSG) device configuration (Fig. 1b) was employed to facilitate S-parameter characterization that is essential for analyzing the insertion loss and isolation at GHz frequencies. Raman and photoluminescence spectroscopy of the CVD-grown MoS_2_ atomic sheets revealed clear evidence of monolayer characteristic^18^ as shown in Fig. 1c and Supplementary Fig. 1.

### Non-volatile resistive switching properties

**Fig. 2:**
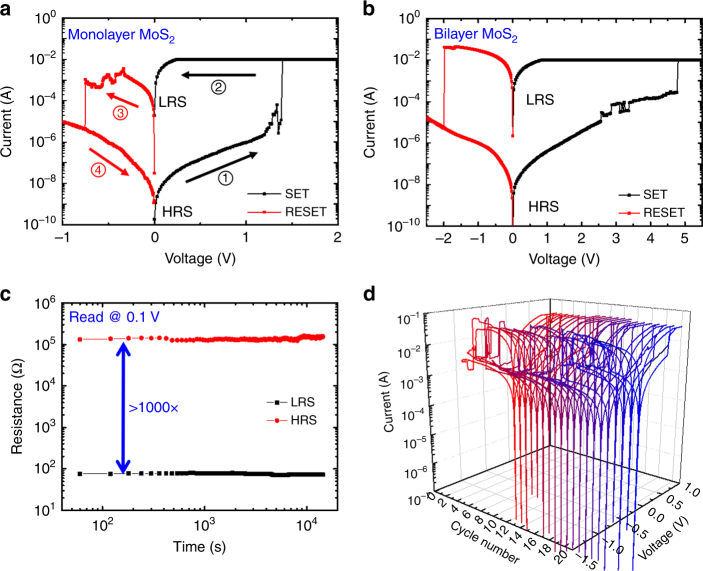
DC switching characteristics of MoS_2_ atomristors. **a** Representative *I*–*V* curve of the bipolar resistance switching effect in a monolayer MoS_2_ RF switch with lateral area of 0.5 × 0.5 μm^2^. Step 1: voltage increases from 0 V. At ~1.4 V, the current abruptly increases to compliance current, indicating a transition (SET) from a high-resistance state to a low-resistance state. Step 2: voltage decreases from 2 to 0 V. The device persists in the low-resistive state. Step 3: voltage decreases from 0 to −1 V. At approximately −0.7 V, the current abruptly decreases, indicating a transition (RESET) from LRS to HRS. Step 4: voltage returns back to 0 V. The device persists in HRS until the next cycle. **b** Representative *I*–*V* curve of the bipolar resistance switching effect in a bilayer MoS_2_ RF switch with lateral area of 0.5 × 0.5 μm^2^ showing similar switching profile. **c** The retention measurement of a monolayer MoS_2_ switch with lateral area of 0.5 × 1 μm^2^ at room temperature revealing stable operation. The HRS and LRS resistance are determined by measuring the current at a small bias of 0.1 V. **d** Typical DC cycling of monolayer MoS_2_ non-volatile resistance switches

Initial measurements focused on DC electrical studies, which were conducted on the non-volatile memory devices consisting of monolayer or bilayer MoS_2_ sandwiched between Au bottom and top electrodes. At the outset, the MoS_2_ atomristors are typically in the high-resistance state until the application of a SET voltage (~1–1.2 V for monolayer), which switches the device to the LRS. Then the switch persists in the LRS until a negative bias is applied to RESET it as shown in Fig. 2a. This distinct *I*–*V* characteristic is known as bipolar switching, and a compliance current is applied during the SET process to protect the device from damage due to excessive current. Ultimately, the compliance current determines the ON-state resistance (Supplementary Fig. 2), a phenomenon attributable to an expansion in the cross-sectional area of the conducting (single) filament or to the creation of multiple filaments^19^. From an RF perspective, such programmable resistance states can be used for electronically controllable attenuators or tunable resistors. Furthermore, ON-state resistance values, <~10 Ω, is critical for low-loss non-volatile RF switch circuits.

Bilayer MoS_2_ MIM devices have a similar bipolar switching property (Fig. 2b) with generally higher switching voltage due to the thicker film^13^. Time-dependent measurements suggest good data retention while maintaining a high ON/OFF ratio (>1000) under ambient conditions (Fig. 2c), which is beneficial for saving power during static periods. The reliability of the monolayer MoS_2_ RF device was tested under DC voltage sweep and showed an endurance of >20 cycles (Fig. 2d) and over 100 cycles in our prior report^13^. The endurance performance is not yet sufficient to meet the tight requirements for RF switching and further research is warranted to engineer the materials and interfaces for increased endurance. For example, endurance can be improved by doping or oxygenation using interface engineering as was previously shown for amorphous carbon resistance change devices, where endurance improved over 10,000 cycles^20^. We are now actively pursuing this partial oxidation route as a method for endurance improvement. However, the monolayer partial oxidation is more challenging than the recent report on high-endurance partially oxidized multilayer MoS_2_^21^; hence, this matter is a subject of ongoing research that will be the focus of a future report. The (limited) statistical distribution of HRS/LRS resistance, SET/RESET voltage, and current over 20 cycles show that the RESET transitions to LRS are more stable than the SET transitions (Supplementary Fig. 3). Furthermore, pulse switching indicates a switching time <30 ns (Supplementary Fig. 4).

### RF performance studies

Toward the goal of switching applications, RF performance studies on the MoS_2_ atomristors were conducted up to 50 GHz using a Keysight vector network analyzer (VNA). To avoid nonlinear effects, the input RF power was set to −20 dBm, which is in the small signal range for S-parameter measurements. In order to obtain precise results and to remove the effects of parasitic impedances arising from the cables and probe station, a standard short-open-load-thru (SOLT) calibration was performed. Subsequently, the intrinsic S-parameter of MoS_2_ MIM devices are obtained after de-embedding the pad and interconnect resistances, using test patterns fabricated on the same substrate (Supplementary Fig. 5)^22^.

**Fig. 3:**
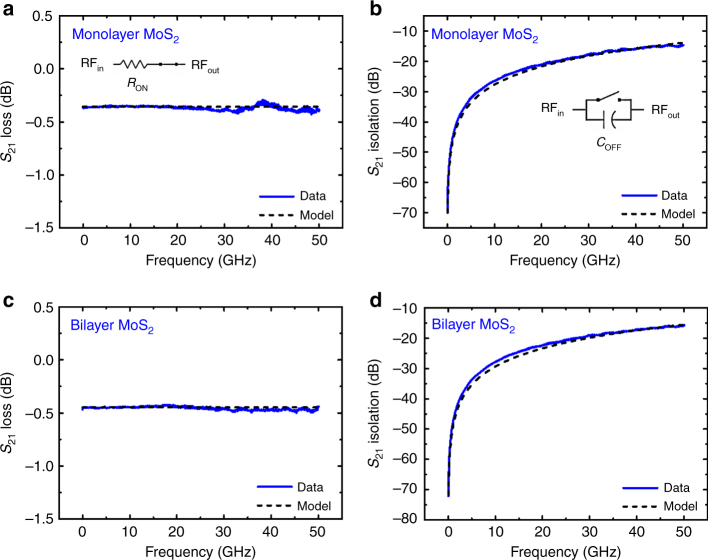
Radio-frequency characterization of MoS_2_ RF switches. **a**, **b** Experimental S(cattering)-parameter data in both the ON-state (insertion loss) and OFF-state (isolation) of an RF switch based on 0.5 × 0.5 μm^2^ monolayer MoS_2_ atomristor. The extracted *R*_ON_ and *C*_OFF_ values are 4.2 Ω and 6.5 fF, respectively. **c**, **d** S-parameter insertion loss and isolation data of an RF switch based on 0.5 × 0.5 μm^2^ bilayer MoS_2_ atomristor. *R*_ON_ is 5.3 Ω and *C*_OFF_ is 5.4 fF. The dashed lines are derived from an equivalent circuit model

The intrinsic experimental RF characteristics of monolayer MoS_2_ switch show promising results of ~0.3 dB insertion loss in the ON-state (Fig. 3a) and isolation <20 dB in the OFF-state (Fig. 3b) at frequencies up to 50 GHz. These results compare favorably with recent memristive RF switches^10^ with the advantage of a simpler and repeatable fabrication process based on a rapidly maturing nanomaterial platform. Bilayer MoS_2_ RF switch shows similar but higher insertion loss (Fig. 3c) and isolation (Fig. 3d) in the ON and OFF states, respectively. The former is understandable due to the longer transport channel. The insertion loss can be reduced by relaxing the compliance current; however, the narrow interconnect feed lines have to be considered carefully to ensure it can handle the current density. In the OFF-state, the normalized average capacitances of monolayer and bilayer RF switches were statistically extracted (Supplementary Fig. 6). RF switches based on 0.5 × 0.5 μm^2^ monolayer and bilayer MoS_2_ atomristors have average capacitances of 28 and 21 fF/μm^2^, respectively. Moreover, monolayer MoS_2_ switches have larger variations due to the stronger sensitivity of the dielectric constant to thickness, strain, interface, and carrier density effects^23–25^. This study highlights the importance of the thickness of the active material for MIM non-volatile RF switches. Monolayer and perhaps bilayer are the most suitable due to low insertion loss. Thicker films of MoS_2_, and by extension TMDs, have an *R*_ON_ that approximately increases with the number of layers^13^ and become excessively lossy for high-performance communication systems.

**Fig. 4:**
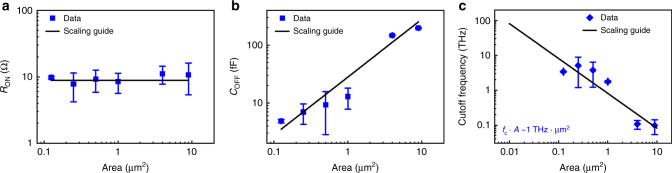
Device scaling performance of the RF switches based on MoS_2_ atomristor. The equivalent lumped element model parameters: **a** ON-state resistance, **b** OFF-state capacitance, and **c** cutoff frequency dependencies on the area. The error bar indicates the standard deviation. The normalized figure of merit, *f*_c_ ∙ *A* ~ 1 THz-μm^2^. While the ON-state resistance is area independent, the OFF-state capacitance is dependent on the lateral area of the device and has a normalized capacitance of ~28 fF/μm^2^. The lines in the figures are area-scaling guides and the slopes of **b**, **c** are based on the statistically averaged capacitance value

Almost two dozen monolayer MoS_2_ atomristors were realized and their area dependence studied for non-volatile RF switches with the results summarized in Fig. 4. A widely used, simple, but accurate lumped element equivalent circuit model (Supplementary Fig. 7 and Supplementary Note 1) was employed to extract the three important performance metrics, *R*_ON_, *C*_OFF_, and *f*_c_^10,26^. In brief, *R*_ON_ is determined from the low-frequency insertion loss in the ON-state and represents the intrinsic loss of the switch while *C*_OFF_ is extracted in the OFF-state and is due to the MIM capacitance that limits the maximum frequency of operation. As expected, the ON-state resistance shows negligible area dependence affirming a one-dimensional filamentary (or bridge) conduction mechanism (Fig. 4a). The lowest achieved *R*_ON_ is about 4 Ω, a favorable value for reduced insertion loss switches. In contrast, *C*_OFF_ is area dependent (Fig. 4b) due to the parallel plate capacitance proportionality relation (*C* ~ *A*) (Supplementary Note 2). The capacitance area data was statistically extracted, *C*_OFF_ ~28 fF/μm^2^. Considering the effective thickness between the electrodes is about a nanometer based on the monolayer thickness and van der Waals gap at the interfaces^27^, the extracted capacitance corresponds to an effective dielectric constant of ~3.2, which is consistent with the significantly reduced value for a monolayer compared to the bulk MoS_2_^28^. The resulting area-dependent statistics of the cutoff frequency FOM is displayed in Fig. 4c. For the experimental set of atomristors with a fabricated area range from 0.125–9 μm^2^, the highest *f*_c_ is ~11 THz. This value is higher or comparable to more mature switching devices, including transistor, MEMS, and PCM RF switches (Supplementary Tables 1 and 2)^4,29–31^, with the added benefit of area frequency scalability by reducing the area to attain higher frequencies without sacrificing insertion loss. The area–frequency expression can be derived from the FOM formula with only *C*_OFF_ showing area dependence, therefore, *f*_c_ ∝ 1/*C*_OFF_ ∝ 1/*A*. As such, *f*_c_ ∙ *A* is a constant, a unique, and a beneficial property for atomristor MIM switches. From the inverse linear fit to the experimental *f*_c_ data (Fig. 4c), *f*_c_ ∙ *A* ~ 1 THz-μm^2^. This is a promising prospect, for example, a 0.01 μm^2^ atomristor switch will afford a cutoff frequency of 100 THz, about an order of magnitude higher FOM than transistor, MEMS, and PCM switches^8,32,33^. Other monolayer semiconductor or insulating TMDs provide an additional degree of design vis-à-vis their material-dependent dielectric constant^34^ that directly determines the MIM capacitance and can enable even higher cutoff frequencies. For higher-frequency operation, the atomristor can be scaled to smaller dimensions <0.1 μm^2^; however, the corresponding scaled metal interconnect lines can fail due to Joule heating from the high currents. As a result, research on thermal management strategies to overcome this issue is underway, specifically, investigation of high thermal conductivity dielectric layers and optimization of the interconnect dimensions.

### RF power handling and self-switching

**Fig. 5:**
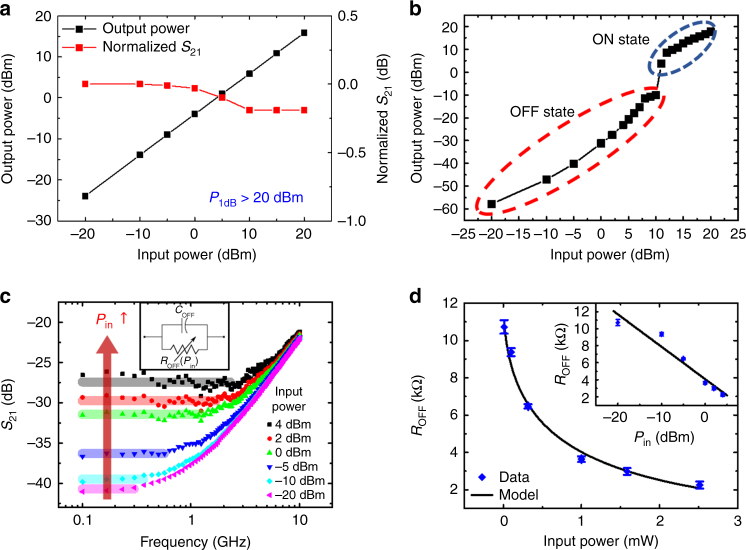
Signal power handling of MoS_2_ RF switches. **a** Representative ON-state *P*_1dB_ and normalized insertion loss measured at 1 GHz in monolayer MoS_2_ RF switch with lateral area of 0.25 × 0.5 μm^2^. **b** OFF-state power handling measured at 1 GHz in monolayer MoS_2_ RF switch with lateral area of 0.5 × 0.5 μm^2^. **c** Transmission spectrum of an OFF-state monolayer MoS_2_ RF switch at different RF input powers. The inset shows an equivalent lumped element circuit model including an OFF-state capacitance (~13 fF) in parallel with an OFF-state variable resistance. **d** OFF-state resistance has an exponentially decreasing dependence on RF input power, which can be modeled as a diode-like voltage-dependent resistance (model line). The error bar indicates the standard deviation

An important performance metric for practical RF switches is the 1 dB compression point (*P*_1dB_)^1,9^, which is an indicator of the maximum input power handling capability of the switch in the ON-state. In order to evaluate this parameter, power-dependent insertion loss measurements were conducted (Fig. 5a), which revealed that output power compression beyond 0.25 dB was not observable at input powers up to 20 dBm, the limit of the commercial VNA source. Hence, we can conclude that the *P*_1dB_ exceeds 20 dBm. More advanced measurements likely using a custom-designed high-power experimental set-up will undoubtedly be needed to precisely measure this power limit.

RF power-induced tunable self-switching is another promising application of MoS_2_ atomristors due to its low-actuation voltage. The feasibility of self-switching was evaluated in an initially OFF device by gradually increasing the RF power from −20 to +20 dBm. It was found that the monolayer device could be switched from the OFF- to ON-state by RF signals at power levels of ~11 dBm (Fig. 5b), which corresponds to about 1.1 V peak voltage and is consistent with the DC switching voltage. This can be a beneficial attribute for wireless switching of the monolayer atomristor that can afford remote-controlled system reconfigurability. On the other hand, we did not find any evidence of RF switching from the ON- to OFF-state. Along the same line, we also investigated the power dependency of monolayer device at a higher frequency (3 GHz), revealing similar self-switching behavior which implies that a wideband of frequencies can be employed for wireless switching (Supplementary Fig. 8). Subsequently, the isolation performance in the OFF-state was analyzed at various input RF power using an equivalent lumped element circuit model (Fig. 5c). The equivalent circuit model has an OFF-state variable resistance paralleled with an OFF-state capacitance. We observed that the OFF-state resistance had an inverse exponential dependence on the root mean square value of the input signal voltage (Fig. 5d). This relation approximately follows the ON-resistance behavior of a PN junction diode (Supplementary Note 3) with an ideality factor of ~7.9 and a saturation current of ~16 μA at 25 mV thermal voltage. On the other hand, although a bilayer MoS_2_ RF switch showed similar behavior for ON-state power dependency, we did not observe self-switching in the OFF-state (Supplementary Fig. 9), a consequence of the higher-actuation voltages for thicker films of MoS_2_. This alludes to the importance of the number of layers as a critical degree of freedom in the design space of MoS_2_ RF switches. Furthermore, higher actuation voltages have also been observed in monolayer atomristors with decreasing lateral area^13^, indicating a strong prospect for improved power handling with dimensional scaling, in addition to the scaling benefits of higher cutoff frequencies discussed earlier.

## Discussion

In summary, we report the detailed investigation of nanoscale non-volatile low-power RF switches based on MoS_2_ atomristors for the first time. The device structure and fabrication process for atomristors are simpler compared to other types of RF switches such as phase-change or MEMS devices due to the lack of a thermal activation or mechanical motion, respectively. In terms of performance, the MoS_2_ switches have low insertion loss and high isolation to 50 GHz, scalable cutoff frequencies beyond 100 THz, and displays good linearity in the ON-state beyond 20 dBm and self-switching in the OFF-state around 11 dBm. Furthermore, atomristors afford low programming voltages and nanoscale dimensions with dimensional-scaling benefits, which combined with the non-volatility is a compelling 2D device concept for facile integration with low-power complementary metal–oxide semiconductor circuits to broaden their functionality for energy-efficient communication systems. Although it is necessary to understand and engineer the interface energetics and ion transport to further advance performance, RF switches based on atomristor could become an attractive candidate for the next-generation reconfigurable communication, connectivity, and IoT systems.

## Methods

### Device fabrication and characterization

The MoS_2_ RF switches were fabricated on a 100 μm-thick Corning Willow glass substrate. The fabrication procedure involved three major steps that defined the bottom electrodes with GSG structures, followed by transferring MoS_2_ atomic sheet and definition of top electrodes. The GSG structure was patterned by EBL. We used e-beam evaporation to create the ground pads and the bottom signal electrode consisting of 2 nm-thick Cr and 60 nm-thick Au. MoS_2_ atomic sheets were then transferred to the fabricated substrate using resist-free polydimethylsiloxane stamp pick-and-place transfer method or poly(methyl methacrylate)-assisted wet transfer method. Then the active region of the MoS_2_ film was defined by EBL and plasma etching. Finally, the top signal electrode was patterned and deposited by using the same fabrication process as BE. The devices were measured on a Cascade Microtech probe station with a Keysight 4156 semiconductor parameter analyzer under ambient conditions. The pulse switching measurement was performed using Keithley Model 4200-SCS with 4225-PMU module. Raman spectroscopy and photoluminescence were measured using a Renishaw in-Via system with a 532 nm wavelength source. Scanning electron microscope images were collected on a ZEISS Neon 40 instrument with the beam energy at 5 kV.

### RF measurements

On-wafer measurements are performed using the Cascade Infinity GSG probes and Keysight E8361C vector network analyzer from 10 MHz to 67 GHz. For small-signal measurements, the RF power was set to a nominal level of −20 dBm. Before actual device measurement, the SOLT calibration was carried out from 10 MHz to 67 GHz using a Cascade 101–190 Impedance Standard Substrate and Cascade WinCal software (Cascade Microtech Inc.)^35^. Scattering parameters were measured for each device in both the ON- and OFF-state, and parameter extraction was carried out to determine the ON- and OFF-state equivalent lumped circuit component models. Power handling was also evaluated in both the ON- and OFF-state by measuring scattering parameters as the devices were gradually exposed to RF power levels from −20 to +20 dBm. See Supplementary Fig. 5 for the detailed de-embedding process.

### Data availability

The data that support the plots within this paper and other finding of this study are available from the corresponding author upon reasonable request.

## Electronic supplementary material

Supplementary Information
