# Supplementary material for: Zero-static power radio-frequency switches based on MoS2 atomristors
Source: Nat Commun. 2018 Jun 28;9:2524. doi: 10.1038/s41467-018-04934-x (PMC6023925; doi:10.1038/s41467-018-04934-x)
Supplement: Supplementary file 1 — Supplementary Information [file 41467_2018_4934_MOESM1_ESM.pdf]

Supplementary Figures

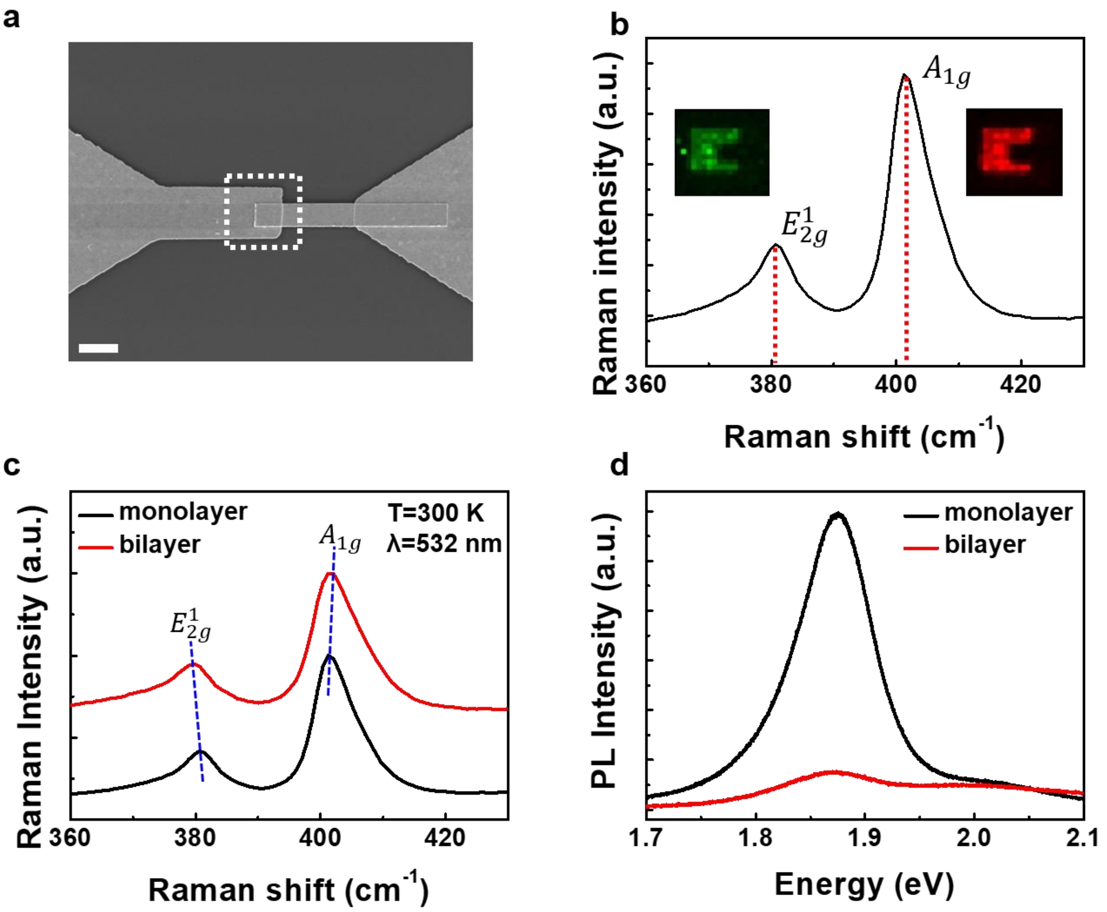

Supplementary Figure 1. MoS_2 Raman and PL characteristics. (a) SEM image of RF switch based on MoS_2 atomristor. The dashed box indicates the area of monolayer MoS_2 sheet. Scale bar, 2 μm. (b) Raman spectra and mapping image of the device. A_{1g} and E^1_{2g} mapping image show similar brightness and geometry. (c, d) Raman spectroscopy and photoluminescence of as-grown monolayer and bilayer MoS_2 films.

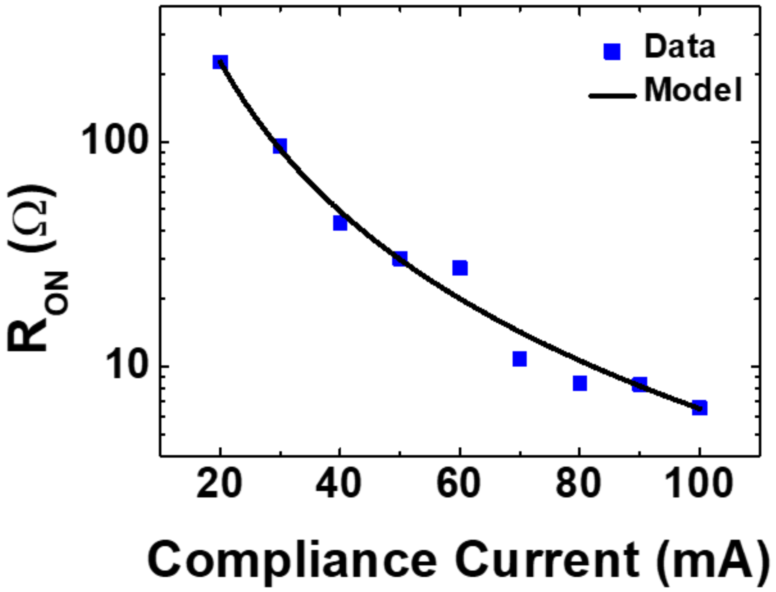

Supplementary Figure 2. ON-state resistance dependency on DC compliance current. ON-state resistance, R_{ON}, and DC compliance current follows $y \sim x^{-n}$ relation with the fitting parameter, n =2.2. Sub-10 Ω resistance can be obtained at LRS.

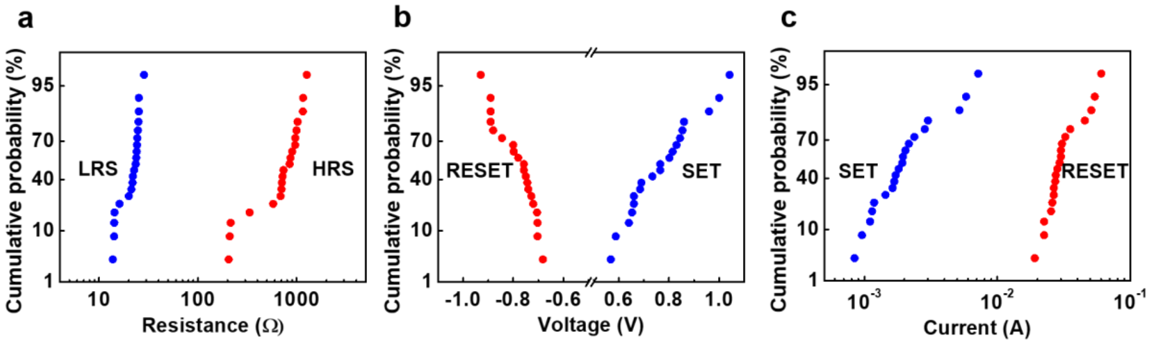

Supplementary Figure 3. Cycling data of the monolayer MoS_2 atomristor. The variability of the single MoS_2 memory device under DC voltage sweep with more than 20 cycles. The overlap area of the device is 5 μm x 5 μm. (a) The distribution of HRS/LRS shows that LRS has less variation compared with HRS. (b) SET/RESET voltage, and (c) SET/RESET current show that the voltage and current at RESET transition are more stable than SET transition.

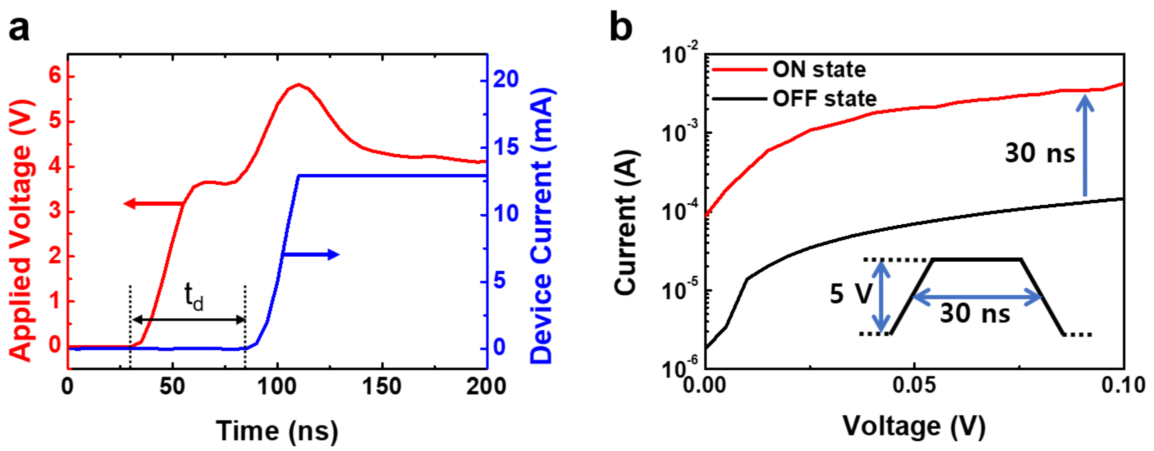

Supplementary Figure 4. Pulse switching of MoS_2 atomristor. (a) Pulse switching of bilayer MoS_2 atomristor from the OFF-state to the ON-state. This switching setup includes waveform capture. The switching or delay time (t_d) in this setup is defined as the time from the start of the applied voltage to the start of the resulting device current,[1] which is about 55 ns. The measured time includes the delay of the cables and the on-chip interconnects and is an upper bound. The overshoot in the applied voltage is due, in part, to the instrument's high-speed impedance matching issues to the non-linear resistance of the switch. (b) Shorter pulses are available from the instrument in the absence of in-situ waveform capture. In this setup, a 30 ns pulse is applied to the MoS_2 atomristor and I-V characteristics are displayed before and after, clearly revealing a switch from the OFF to ON state. Therefore, we can conclude that the intrinsic switching time is less than 30 ns.

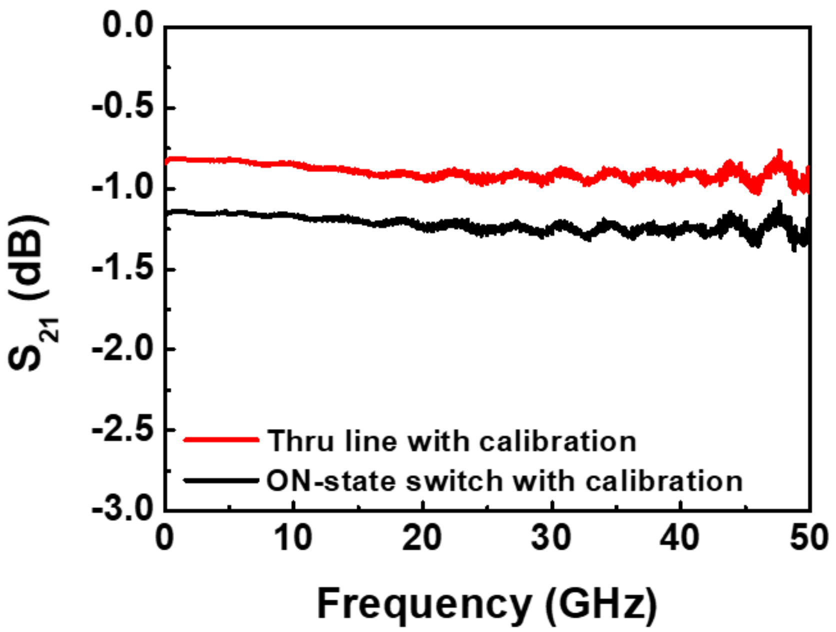

Supplementary Figure 5. S_{21} Comparison of ON-state device to Thru line. The ON-state device S_{21} is approximately 0.4 dB greater than that of the Thru line. An SOLT calibration using a Cascade 101-190 Impedance Standard Substrate (ISS) and the associated WinCal software were employed for these measurements.[2] The calibration establishes the reference point to the ground-signal-ground probe tips. In order to obtain the corrected switch insertion loss, a Thru line having the same line shape and on the same substrate as the switch (but without MoS_2) was separately measured and afterward used to de-embed the parasitic losses from the ON-state switch. Essentially, this enables extraction of the intrinsic loss of the MoS_2 RF switch in the ON-state. For the OFF-state condition, the same calibration is used, however, no de-embedding was applied since we determined that the parasitic effects were negligible in the measured frequency range, that is, the loss and capacitance were predominantly arising from the MoS_2 MIM switch.

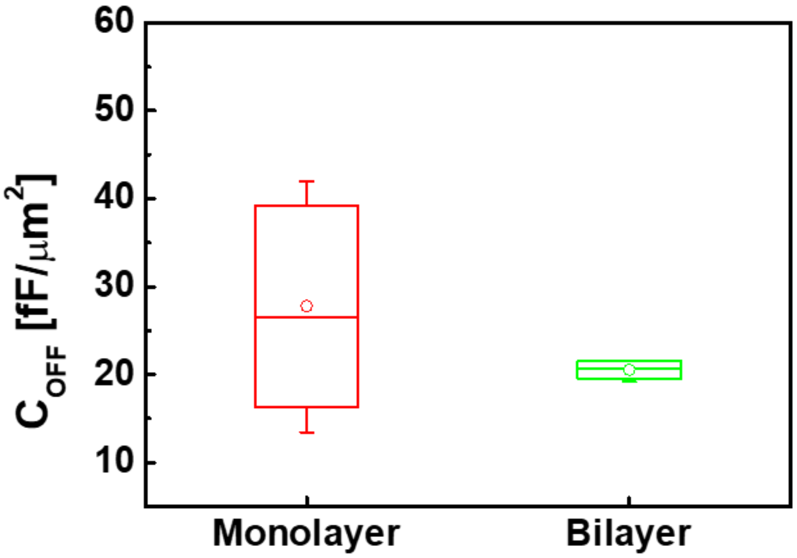

Supplementary Figure 6. Comparison of monolayer and bilayer MoS_2 switch OFF-state capacitances. RF switches based on 0.5×0.5 μm^2 monolayer and bilayer MoS_2 atomristor shows 28 $fF/\mu m^2$ and 21 $fF/\mu m^2$ average capacitance values. For the box plot, the middle circle indicates the mean. The bottom and top of the box are one standard deviation above and below the mean of the data, and the line inside the box is the median. The ends of the whiskers represent the minimum and maximum of all the data. The larger variability of the monolayer MoS_2 switch is attributed, in part, to its larger sensitivity to electrical, thermal, chemical and mechanical interfacial effect compared to the thicker bilayer. This variability, an indicator of the infant state of atomristor devices, requires further research for practical applications.

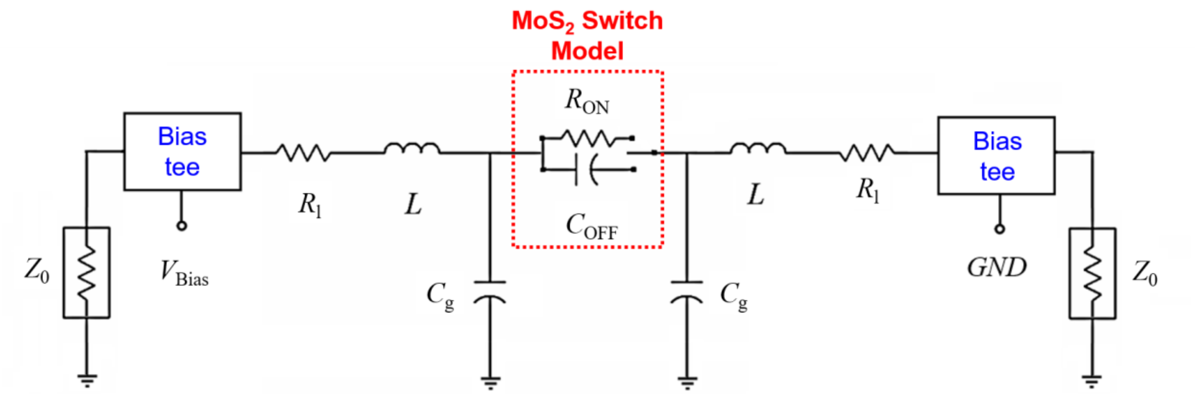

Supplementary Figure 7. Equivalent lumped element circuit model. The figure shows the equivalent RF switch circuit model. The model includes the characteristic impedance, Z_0; a line resistor, R_l; a line inductor, L; a switch series OFF-state capacitor, C_{OFF} or an ON-state resistance, R_{ON}; and a shunt coupling capacitor to ground, C_{g}.

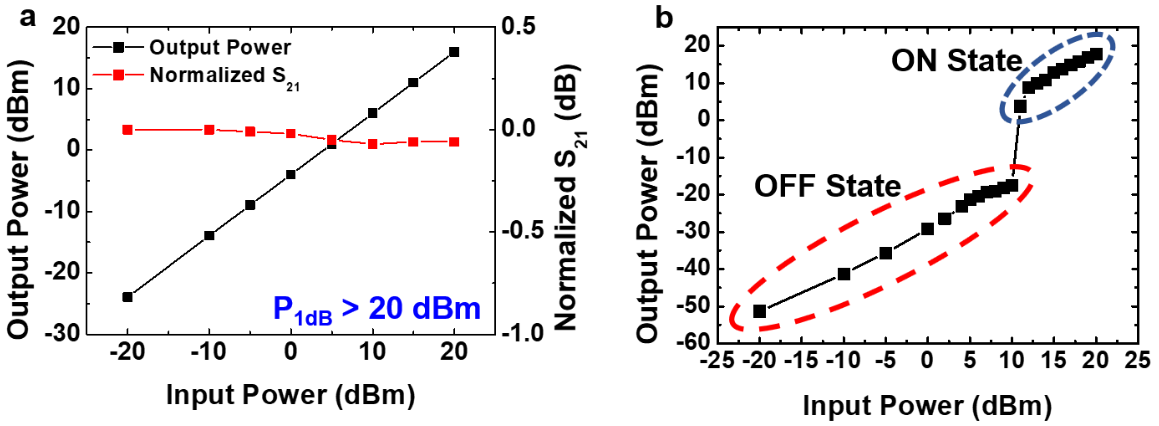

Supplementary Figure 8. Signal power handling of monolayer MoS_2 RF switch at 3 GHz. (a) Representative P_{1dB} and normalized loss measured in the ON state at 3 GHz in monolayer MoS_2 switch with lateral area of 0.25×0.5 μm^2. (b) Representative OFF-state RF power handling measured at 3 GHz in monolayer MoS_2 switch with lateral area of 0.5 × 0.5 μm^2.

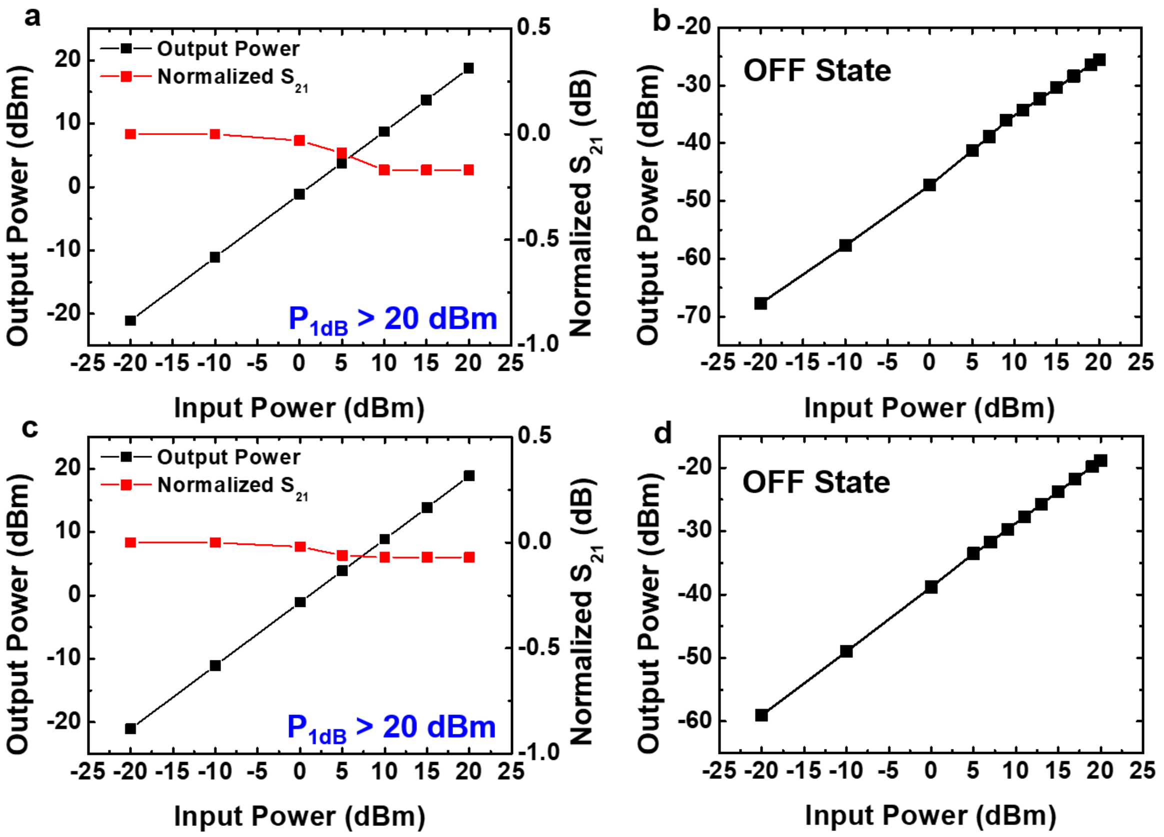

Supplementary Figure 9. Signal power handling of bilayer MoS_2 RF switch. The figure shows representative ON-state P_{1dB}, normalized insertion loss and OFF-state RF power capability at (a, b) 1 GHz, (c, d) 3 GHz in bilayer MoS_2 switch with lateral area of 0.5×0.5 μm^2.

Supplementary Tables

Supplementary Table 1. Comparison table of 3x3 μm^2 GeTe phase-change and MoS_2 RF switches

Materials	GeTe	MoS_2 (This work)
**Area [μm^2]**	3 x 3	3 x 3
**Thickness [nm]**	≥ 100	~1
**C_{OFF} [fF]**	20	190
**R_{ON} [Ω]**	1.75	5.3
**Switching Temperature**	~480 K	Room temperature
**Switching Speed [ns]**	500-20,000	< 55
**Switching Voltage [V]**	1-4	0.5-1.5

Generally, the device size of the phase change switches from chalcogenide compounds are larger than MoS_2 RF switches. Shim et al.[3] reported the measured ON-state resistance and OFF-state capacitance of a 3x3 μm^2 lateral GeTe RF switch. Here, we compared the switching temperature, speed, voltage, and thickness of the same area GeTe phase-change switch and MoS_2 aromristor RF switch.

Supplementary Table 2. RF switch comparison table.

Feature\Device	Transistor Switch [4]	MEMS Switch [5]	VO_2 Phase-change Switch [6]	GeTe Phase-change Switch [3]	GeTe Phase-change Switch [7]	Memristive switch [8]	2D Switch (this effort)
**Material**	SOI	Au-Ru contact array	VO_2 via array	GeTe	GeTe	Ag-Au/Ti	CVD MoS_2
**Non-volatility**	No	No	No	Yes	Yes	Yes	Yes
**Control Voltage**	2.5 V	~65 V	~1.5 V	1-4 V	3.5 V	3V	~0.5 – 1.5V
**ON-Resistance**	0.8 Ω mm	1.4 Ω	6 Ω	~1.75 Ω	1.7 Ω	3.6 Ω	4 Ω
**OFF-capacitance**	312 fF/mm	30 fF	1 fF	~15 fF	5.4 fF	1.4 fF	3 fF
**Cutoff frequency**	0.63 THz	3.8 THz	26.5 THz	~ 6 THz	17 THz	35.2 THz	13.2 THz
**Operating Environment**	Ambient condition	Hermetic packaging	Ambient condition	Heater needed	Heater needed	Ambient condition	Ambient condition
**Switching time**	-	2.2 us	25 ns	0.5-20 us	< 0.5 us	-	< 30 ns
**Dimension (single device W x L)**	180nm node	300 um x 24 um	1 um x 100 nm	3 um x 3 um	800 nm x 500 nm	110 nm x 35 nm	250 nm x 500 nm

Supplementary Notes

Supplementary Note 1. S-parameter equation from equivalent lumped element circuit model

As reported by the T-equivalent circuit model, S_{21} of the circuit from the Supplementary Figure 7 is given by

$$S_{21} = \frac{2}{2 + (Z_0 + Z_2 + Z_2)/Z_3 + (Z_1 + Z_2 + \frac{Z_1 Z_2}{Z_3})/Z_0} \tag{1}$$

where

$$Z_1 = R_\mathrm{l} + j\omega L \tag{2}$$

$$Z_2 = \begin{cases} 1/j\omega C_\mathrm{OFF} \\ R_\mathrm{ON} \end{cases} \tag{3}$$

$$Z_3 = \frac{1}{j\omega C_\mathrm{g}} \tag{4}$$

ω is the angular frequency.

The switch capacitance, C_OFF, is an important factor that affects the isolation of the switch in the OFF-state of the switch. OFF-state isolation of the switch can be approximately expressed as

$$S_{21,\,\mathrm{Isolation}} \approx \frac{2}{2 + (\frac{1}{j\omega C_\mathrm{OFF}})/Z_0} \tag{5}$$

In order to subtract the effects of the line resistor, R_l, we measured the scattering parameters of the same device structure without 2D material and de-embedded it from the ON-state measurement. Therefore, ON-state insertion loss can be simplified as

$$S_{21,\,\mathrm{Loss}} \approx \frac{2}{2 + R_{ON}/Z_0} \tag{6}$$

Supplementary Note 2. Physical modeling of the OFF-state capacitance

As we showed in Supplementary Figure 6, the statistically extracted OFF-state capacitance values of monolayer and bilayer MoS_2 RF switches follows the intuitive expectation of thickness dependence. The experimentally deduced average values can be related to a lumped element model based on the physical geometry. From the parallel-plate capacitor model, the area-normalized capacitance is given by

$$\boldsymbol{C = \frac{\varepsilon_0 k}{d}\ [\mathrm{fF}/\mu m^2]} \tag{7}$$

From Song et al., the effective thickness of monolayer and bilayer MoS_2 are 1 and 1.6 nm, which includes van der Waals interface gaps.[9] Using these reasonable thicknesses and the experimental average OFF-state capacitances, we employed the parallel-plate equation to estimate the effective dielectric constants, which were found to be 3.2 and 3.8 for monolayer and bilayer, respectively. It is notable that the estimated dielectric constants are within 15 % of the statistical range reported in Chen et al.[10] which uses a different experimental technique to estimate the dielectric constant. As such, we can conclude that our experimental interpretation is consistent with thickness-dependent expectation and prior literature report.

Supplementary Note 3. OFF-state resistance of RF switch modeled by PN junction diode equation.

From the Shockley diode equation,

$$I = I_S\,(e^{\frac{V_D}{nV_T}} - 1) \tag{8}$$

I is the diode current, I_S is the reverse bias saturation current (or scale current), V_D is the voltage across the diode, V_T is the thermal voltage kT/q (Boltzmann constant times temperature divided by electron charge), and n is the ideality factor introduced to model a slower rate of increase than predicted by the ideal diode law.

$$V_T = \frac{kT}{q} \tag{9}$$

$$r_D = \frac{dI}{dV} \approx \frac{nV_T}{I} = \left(\frac{nV_T}{I_S}\right) e^{-\frac{V_D}{nV_T}} \tag{10}$$

$$r_D \propto e^{-V_D} \tag{11}$$

From the input RF power dependent R_{OFF} fitting, R_{OFF} exponentially decreases as the input voltage increases. This dependence can be fitted to the diode equation,

$$R_{OFF} \cong \left(\frac{nV_T}{I_S}\right) e^{-\frac{V_{RMS}}{nV_T}} \tag{12}$$

$$nV_T = 198.85\ mV \tag{13}$$

$$\left(\frac{nV_T}{I_S}\right) = 12.39\ k\Omega \tag{14}$$

We can extract the parameters n, I_S

$$n = 7.9\ @\ V_T = 25\ mV \tag{15}$$

$$I_S = 16.04\ \mu A \tag{16}$$

Supplementary References

1. Tsuruoka T, Hasegawa T, Valov I, Waser R, Aono M. Rate-limiting processes in the fast SET operation of a gapless-type Cu-Ta2O5 atomic switch. *AIP Adv.* **3**, 032114 (2013).
2. Cascade Microtech Application Note. On Wafer Vector Network Analyzer Calibration and Measurements. https://www.cascademicrotech.com/files/ONWAFER.pdf, (1997).
3. Shim Y, Hummel G, Rais-Zadeh M. RF switches using phase change materials. 2013 IEEE 26th International Conference on Micro Electro Mechanical Systems (MEMS), 237-240 (2013).
4. Botula A, *et al.* A thin-film SOI 180nm CMOS RF switch technology. IEEE Topical Meeting on Silicon Monolithic Integrated Circuits in RF Systems, SiRF'09, 1-4 (2009).
5. Stefanini R, Chatras M, Blondy P, Rebeiz GM. Miniature MEMS switches for RF applications. *J. Microelectromech. Syst.* **20**, 1324-1335 (2011).
6. Madan H, *et al.* 26.5 Terahertz electrically triggered RF switch on epitaxial VO2-on-Sapphire (VOS) wafer. 2015 IEEE International Electron Devices Meeting (IEDM), 9.3. 1-9.3. 4 (2015).
7. Léon A, *et al.* In-depth caracterisation of the structural phase change of Germanium Telluride for RF switches. 2017 IEEE MTT-S International Microwave Workshop Series on Advanced Materials and Processes for RF and THz Applications (IMWS-AMP), 1-3 (2017).
8. Pi S, Ghadiri-Sadrabadi M, Bardin JC, Xia Q. Nanoscale memristive radiofrequency switches. *Nat. Commun.* **6**, 7519 (2015).
9. Song J-G, *et al.* Controllable synthesis of molybdenum tungsten disulfide alloy for vertically composition-controlled multilayer. *Nat. Commun.* **6**, 7817 (2015).
10. Chen X, *et al.* Probing the electron states and metal-insulator transition mechanisms in molybdenum disulphide vertical heterostructures. *Nat. Commun.* **6**, (2015).
